# Development of solution-gated graphene transistor model for biosensors

**DOI:** 10.1186/1556-276X-9-71

**Published:** 2014-02-11

**Authors:** Hediyeh Karimi, Rubiyah Yusof, Rasoul Rahmani, Hoda Hosseinpour, Mohammad T Ahmadi

**Affiliations:** 1 Centre for Artificial Intelligence and Robotics, Universiti Teknologi Malaysia, Jalan Semarak, Kuala Lumpur 54100, Malaysia; 2 Malaysia-Japan International Institute of Technology (MJIIT), Universiti Teknologi Malaysia, Jalan Semarak, Kuala Lumpur 54100, Malaysia; 3 Department of Biotechnology Industry, Faculty of Bioscience and Medical Engineering, Universiti Teknologi Malaysia, 81310 Johor, Malaysia; 4 Computational Nanoelectronic Research Group, Faculty of Electrical Engineering, Universiti Teknologi Malaysia, Jalan Semarak, Johor 81310, Malaysia; 5 Nanotechnology Research Center Nanoelectronic Group, Physics Department, Urmia University, Urmia 57147, Iran

**Keywords:** Graphene, DNA hybridization, Optimization, Solution-gated field effect transistor, Single-nucleotide polymorphism, Particle swarm optimization

## Abstract

The distinctive properties of graphene, characterized by its high carrier mobility and biocompatibility, have stimulated extreme scientific interest as a promising nanomaterial for future nanoelectronic applications. In particular, graphene-based transistors have been developed rapidly and are considered as an option for DNA sensing applications. Recent findings in the field of DNA biosensors have led to a renewed interest in the identification of genetic risk factors associated with complex human diseases for diagnosis of cancers or hereditary diseases. In this paper, an analytical model of graphene-based solution gated field effect transistors (SGFET) is proposed to constitute an important step towards development of DNA biosensors with high sensitivity and selectivity. Inspired by this fact, a novel strategy for a DNA sensor model with capability of single-nucleotide polymorphism detection is proposed and extensively explained. First of all, graphene-based DNA sensor model is optimized using particle swarm optimization algorithm. Based on the sensing mechanism of DNA sensors, detective parameters (*I*_ds_ and *V*_gmin_) are suggested to facilitate the decision making process. Finally, the behaviour of graphene-based SGFET is predicted in the presence of single-nucleotide polymorphism with an accuracy of more than 98% which guarantees the reliability of the optimized model for any application of the graphene-based DNA sensor. It is expected to achieve the rapid, quick and economical detection of DNA hybridization which could speed up the realization of the next generation of the homecare sensor system.

## Background

With the discovery of graphene, a single atomic layer of graphite, material science has been experiencing a new path in biomedical applications, due to its fascinating properties [1]. Graphene possess extraordinary physical properties, such as a unique electronic band structure, extremely high carrier mobility, biocompatibility and well-known two-dimensional (2D) structure exposing every atom of graphene to the environment [1-3]. It is demonstrated that the high sensitivity of graphene to the charged analytes (ions, DNA, cells, etc.) or an electric field around it renders graphene an ideal material for high-performance sensors. In the last 5 years, there has been an increasing amount of literature on solution-gated field effect transistors (SGFETs) as useful candidates for chemical and biological sensors [4,5]. The interface between nanomaterials and biosystems is emerging as one of the most interesting areas of intense research [6]. Recent advances and key issues for the development of DNA sensors to bridge the knowledge to clinical detection of DNA hybridization emerged as a promising means of diagnostic prediction in genetic research [7,8]. The aim of this paper is to provide a possibility of having more sensitive and sequence-selective DNA biosensors by developing the SGFETs analytical model for electrical detection of DNA molecules [9,10]. Graphene layer is selected as a sensing template because of its large surface-to-volume ratio which guarantees better physical adsorption of DNA due to more accessible contact, compared with other carbon materials [11].

**Figure 1:**
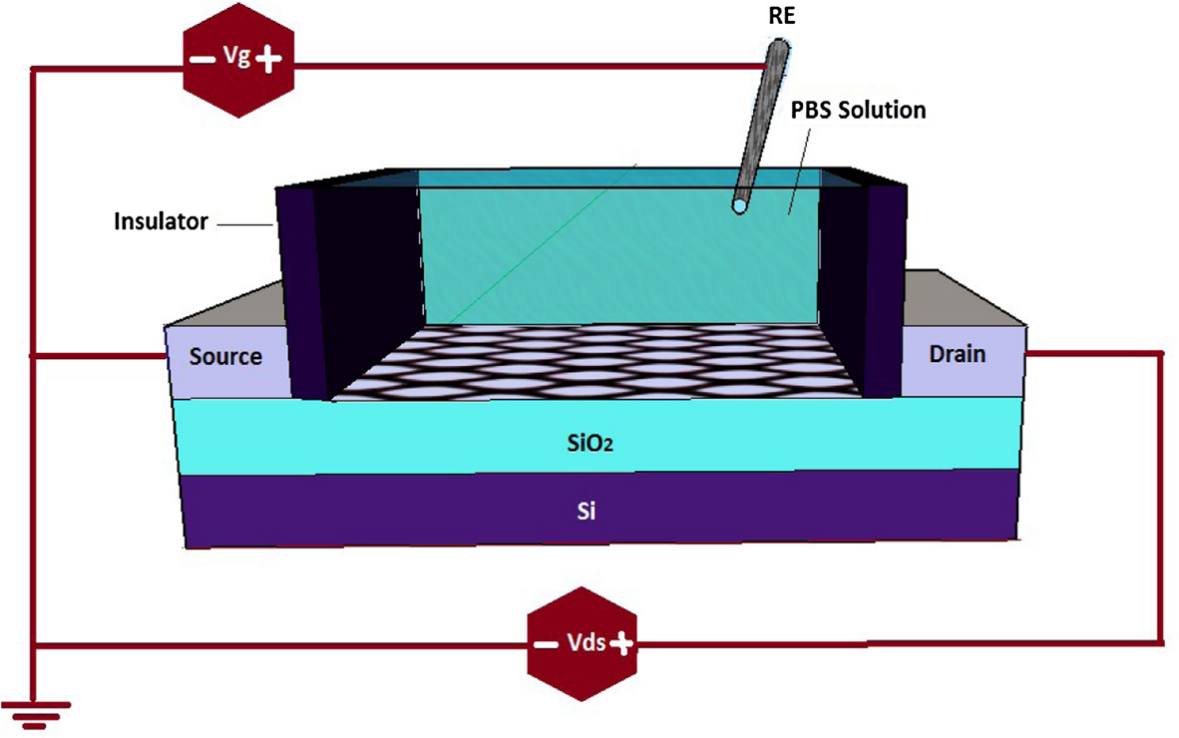
Schematics of DNA sensor structure.

Several numbers of research on the basic of field effect devices for DNA detection have been published in recent years. There are different configurations of DNA sensors such as electrolyte-silicon (ES) structures, depletion and enhancement-mode field effect transistor (FET), with or without a reference electrode [1,12-20]. The focus of this theoretical study will be on developing the DNA sensor-based graphene nanomaterials which have become extremely important for diagnosis and treatment of the gene-related diseases [21,22]. As depicted in Figure 1, SGFET-based DNA sensor structure consists of a 300-nm SiO_2_ layer as a back gate dielectric and a doped silicon substrate used as the back gate has been proposed [2]. Graphene layer as a conducting channel connected to the source and drain electrodes. The possibility of having channels that are just one atomic layer thick is perhaps the most attractive feature of graphene for transistors [23]. An Ag/AgCl wire was inserted into the solution chamber and acted as the gate electrode of a SGFET which controls the current along the graphene sheet between the two electrodes [24,25]. The DNA sensors were exposed to a phosphate buffer solution (PBS) containing the DNA molecules.

It is noteworthy to explain the DNA adsorption effect on nanomaterials of graphene surface as well as the proposed model. In graphene, the electronic transport takes place by hopping along *π* orbitals which is due to the s*p*^2^ hybridized covalent bonds that held the carbon atoms together, while each of them can participate in some kind of bonding with adsorbates [26]. Theoretical data suggest that the bonding between the DNA bases and the carbon atoms is a kind of van der Waals (vdW) bonding (*π*-*π* stacking) [27,28]. Since the DNA molecules have the negative charges, therefore, it could be expected that the adsorption of DNA molecules on graphene surface would directly modulate the drain current of the SGFET device [29,30]. Based on the detection mechanism, we recently proposed an analytical model for the detection of DNA molecules in which the DNA concentration was modelled by a gate voltage [2].

Although there are lots of works presented on the experimental progress, however, far too little attention has been paid to the detection mechanism quantitatively. For supporting this, modelling and simulation using partial differential equations (PDE) play a critical role in determining the current-voltage characteristics, sensitivity and the behaviour of the sensing devices exposing to DNA molecules. Our proposed model is capable of performing the electrical detection of DNA molecules by modelling the conductance of the graphene sheets. Based on the sensing mechanism inspired by the experiment to investigate the effect of DNA adsorption on graphene, DNA concentration as a function of gate voltage is assumed and sensing factor (*α*) is defined. High carrier mobility reported from experiments in the graphene leads to assume a completely ballistic carrier transportation in the graphene [31]. Subsequently, FET modelling was employed to obtain relevance between current versus voltage of gate sensor. The DNA concentration model is employed as a function of gate voltage and the ideal current-voltage relation for the n-channel FET in the non-saturation region from reference [32] is obtained as:

(1) $$\begin{array}{ll} I_{d}= & \frac{3q^{2}{\left(3\pi a^{3}t^{3}k_{B}T\right)}^{0.5}}{\text{hL}}\left[{ȷ}_{-0.5}(\eta )+{ȷ}_{-0.5}(-\eta )\right]\\ & \times \left(\frac{\alpha }{F}V_{\text{gs}(\text{without DNA})}-V_{t}\right) \end{array}$$

Where *q* is the electron charge, *a* = 1.42Å denotes carbon-carbon (C - C) bond length, *t* = 2.7 eV is the nearest neighbour C - C tight binding overlap energy, *k*_*B*_ is the Boltzmann’s constant, *T* represents temperature and *h* is the Planck’s constant. *L* shows the length of conducting channel, *V*_gs_ donates the gate-source voltage and *V*_t_ refers to the threshold voltage. Furthermore, *ȷ*_-0.5_(*η*) and *ȷ*_-0.5_(-*η*) are the Fermi-Dirac integrals of orders -0.5 which can be solved numerically. Its value depends on *η* which measures the location of the Fermi level with respect to the conduction band edge. The Fermi-Dirac distribution function has different forms in degenerate and non-degenerate states which are attributed by (*η* ≫ 0) and (*η* ≪ 0), respectively [5,32]. *α* is DNA sensing factor and different concentration of DNA molecules were presented in the form of *F* parameter. Thus, the DNA molecules adsorbed on graphene surface by iteration method was modelled as

(2) $$\alpha =A\times F^{2}+B\times F+C$$

*A* = 13, *B* = 50 and *C* = 4,070 are the parameters calculated based on the extracted data. The current-voltage characteristic of SGFET according to the proposed model of DNA sensor using nanostructured graphene layer is obtained as:

(3) $$\begin{array}{ll} I_{d}= & \frac{3q^{2}{\left(3\pi a^{3}t^{3}k_{B}T\right)}^{0.5}}{\text{hL}}\left[{ȷ}_{-0.5}(\eta )+{ȷ}_{-0.5}(-\eta )\right]\\ & \times \left(\frac{13F^{2}+50F+4070}{F}V_{\text{gs}(\text{without DNA})}-V_{t}\right) \end{array}$$

It is concluded that the sensor model with the suggested parameters represents the same trend as experimental data [2,6]. Since the values of the parameters *A*, *B* and *C* in Equation 2 were calculated based on trial and error, there is necessity of a methodological approach for obtaining a viable and accurate model which is reliable for being used in different applications of the graphene-based DNA sensor. To this purpose, an evolutionary algorithm (EA) called particle swarm optimization (PSO) is used for optimizing the mathematical model shown in Equation 1. The PSO technique is widely used in optimizing different sorts of problems including fine materials, medical science, control theory, energy issues, etc. [33-36]. The important facts that make PSO popular among the researchers are its fastness, avoiding from being trapped in the local optima, and the capability of being employed in any type of optimization problems [37-40].

## Methods

### Particle swarm optimization overview

The PSO is a swarm-based optimization algorithm which is classified as a metaheuristic optimization algorithm. The idea of the PSO rises from the movement of a bird flock which was first introduced by Kennedy and Eberheart [41-45]. The aim of employing PSO algorithm in this study, is to find the best possible values for *A*, *B* and *C* parameters in Equation 2 which leads to have a more accurate DNA sensor model with better *I*-*V* characteristic. Each particle at each step is supposed to return a set of three values with respect to *A*, *B* and *C* parameters. Afterwards, these values must be evaluated using a proper fitness function. During the optimization process, the values of *A*, *B* and *C* parameters change, until we can get the best possible solutions.

The movement velocity of each particle is updated regularly, at each step. The location and velocity of the *i*th particle at *k*th step are shown in Equations 4 and 5, respectively.

(4) $$X_{i}^{k+1}=X_{i}^{k}+V_{i}^{k+1}$$

(5) $$V_{i}^{k+1}=W\times V_{i}^{k}+c_{1}\times r_{1}({\text{Gbest}}^{k}-X_{i}^{k})+c_{2}\times r_{2}({\text{Pbest}}_{i}^{k}-X_{i}^{k})$$

*i* = 1, 2, …, nop (number of particles); *k* = 1, 2, …, *k*_max_ (maximum iteration number) where *i* is the particle number; *k* is the iteration number; *W* refers to the inertia weight coefficient which is decreased continuously from 1.2 to 0.5, *r*_1_ and *r*_2_ are random values between 0 and 1, *c*_1_ and *c*_2_ are acceleration coefficients and set to be equal to 2, $X_{i}^{k}$ denotes the position and $V_{i}^{k}$ is the velocity of particle *i* at iteration *k*.

**Figure 2:**
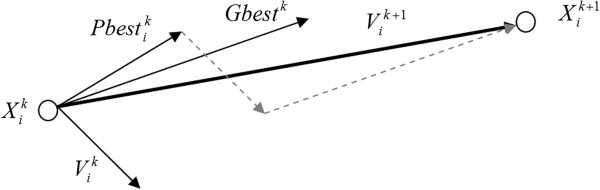
**PSO algorithm.** A simple diagram for movement of a sample particle in PSO.

There are some social parameters that lead the swarm to the global optimum of the search space which are personal best (Pbest) and global best (Gbest). There is one Pbest for each particle which is the best location experienced by it, while Gbest is the best global optimum point found by the swarm. A simple diagram of the movement of a particle is shown in Figure 2. The number of particles in the swarm is considered as 200 which iterate for 300 runs.

A fitness function must be defined for evaluating the particles at each step. Therefore, there is a fitness value for each particle at each step. In this study, the chosen fitness function is shown in Equation 6 which calculates an error value between the real and modelled data.

(6) $$\text{Fitness function}=\psi_{i}=\overset{\max}{\underset{k=1}{\sum }}{\left({Î}_{i}(k)-I(k)\right)}^{2}$$

where *I*(*k*) is the experimental waveform of the DNA sensor, ${Î}_{i}(k)$ represents the value of the modelled waveform for particle *i* and *ψ*_*i*_ is the fitness value for the *i*th particle. Obviously, the lowest fitness value represents the most fitted curve which is desired for a reliable DNA sensor model.

## Results and discussion

### Results of optimization for DNA sensor model

The parameters to be optimized in this model were *A*, *B* and *C* in Equation 2 which create a solution space of four dimensions with three variables and one function known as fitness function. The best results obtained out of 20 runs are shown in Table 1 which introduce the lowest fitness values.

**Table 1:** The best values of the optimizing parameters over the 20 runs

**The best fitness value obtained**	**Optimized value for A**	**Optimized value for B**	**Optimized value for C**
6.742e-07	2.138e10	8.9921e9	-5.680e3

**Figure 3:**
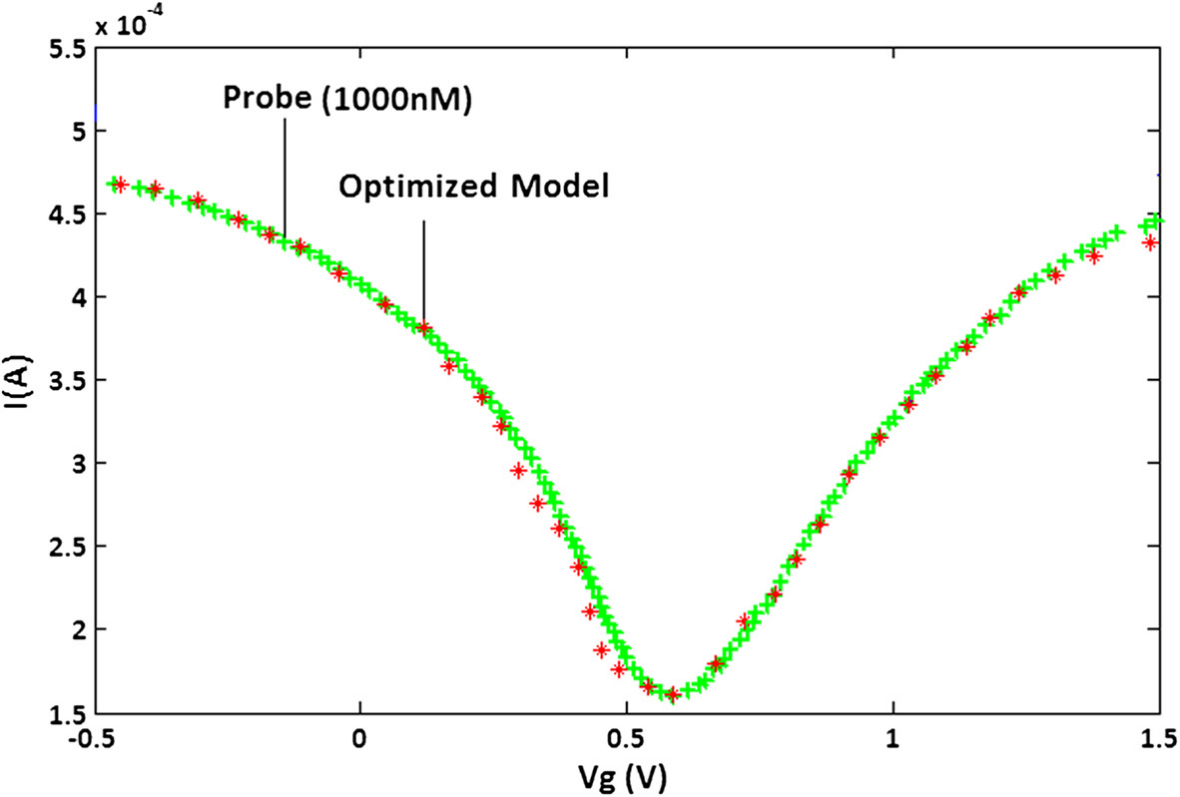
**DNA sensor characteristics.** The experimental and optimized model waveforms for DNA sensor in the presence of probe DNA.

The experimental waveform of the DNA sensor is used for obtaining the optimized values for parameters *A*, *B* and *C*. The optimized model and the experimental waveforms are shown in Figure 3.

The mean absolute percentage error (MAPE) index is used to assess the quality of the modelled waveform (see Equation 7).

(7) $$\text{MAPE}=\frac{1}{n}\overset{n}{\underset{k=1}{\sum }}|\frac{Î(k)-I(k)}{I(k)}|$$

The optimized model is evaluated using the MAPE index for different concentrations of the DNA sensor. Table 2 shows the accuracy of the proposed optimized model for six different concentrations of the DNA sensor covering a range from 0.01 to 500 nM. The lowest accuracy obtained is 98.46% for the concentration of 0.01 nM while the highest accuracy is 99.41% belonging to the concentration of 100 nM. Overall, the accuracy of more than 98% represents an overall error of less than 2% which is quite acceptable for the optimized model.

**Table 2:** **The MAPE value for different concentrations of DNA sensor (*F*)**

**Concentration *F *** **(nM)**	**MAPE value (%)**	**Accuracy based on MAPE (%)**
*F* = 0.01	1.54	98.46
*F* = 0.1	0.90	99.10
*F* = 1	1.03	98.97
*F* = 10	0.77	99.23
*F* = 100	0.59	99.41
*F* = 500	0.93	99.07

In the next section, it is demonstrated that the optimized model of solution-gated graphene-based DNA sensors can be utilized for electrical detection of DNA hybridization application.

### DNA hybridization detection using the optimized model

**Figure 4:**
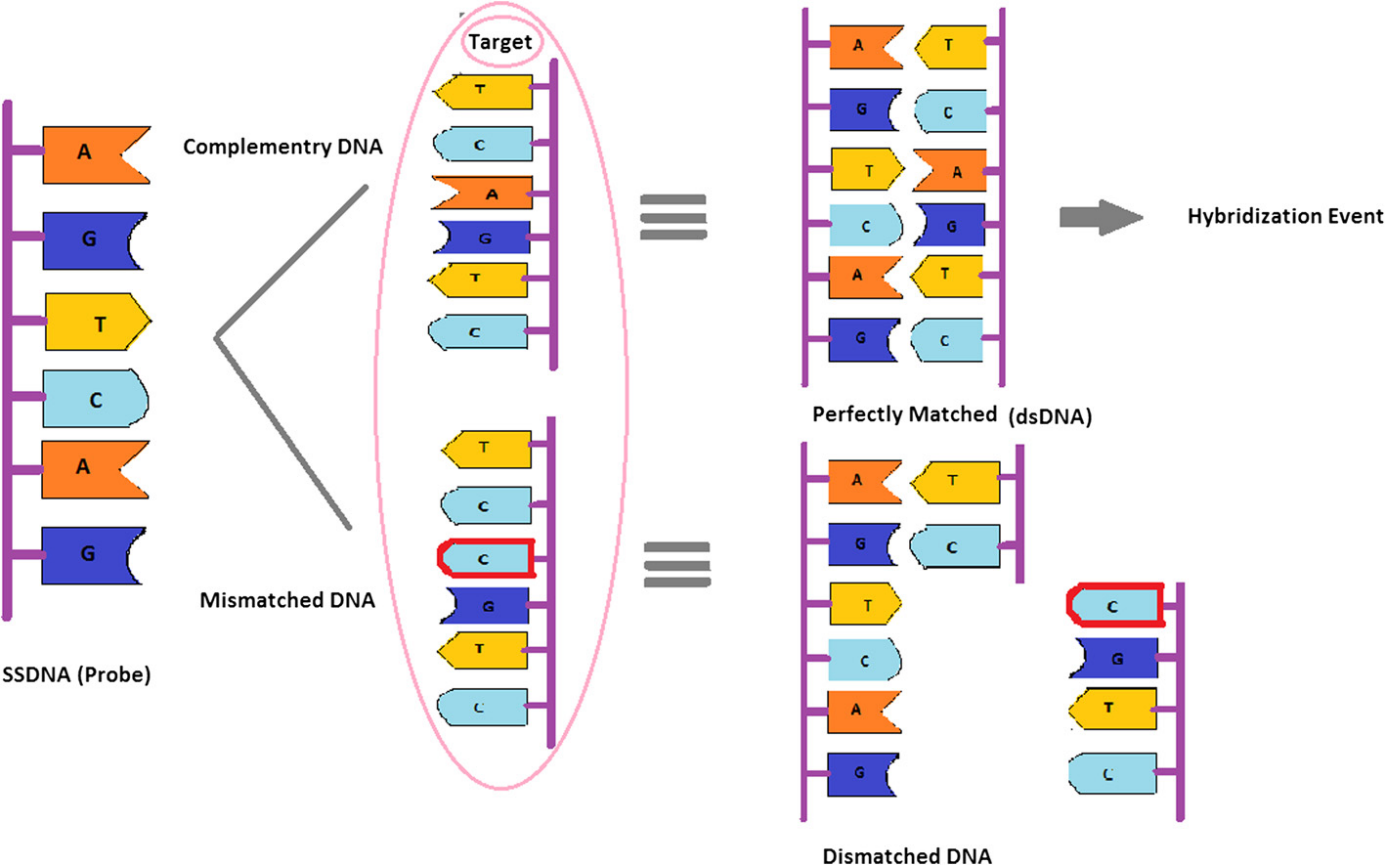
Schematic of DNA hybridization event.

The detection of DNA hybridization has been a topic of central importance owing to a wide variety of applications such as diagnosis of pathogenic and genetic disease, gene expression analysis and the genotyping of mutations and polymorphisms [46,47]. Technologies in DNA biosensing [48] have received special appeal not only for their low cost and simplicity but also for their ultimate capabilities in detecting single-nucleotide polymorphisms (SNP) which have been correlated to several diseases and genetic disorders such as Alzheimer and Parkinson diseases. The DNA hybridization event is the basis of many existing DNA detection techniques. In DNA hybridization as depicted in Figure 4, the target, unknown single-stranded DNA (ssDNA), is identifid and formed by a probe ssDNA and a double-stranded (dsDNA) helix structure with two complementary strands. It is believed that, in the presence of a mixture of diverse non-complementary nucleic acids, the hybridization reaction is known to be extremely efficient and specific. The basis for the high specificity of the biorecognition process is the uniqueness of complementary nature of this binding reaction between the base pairs, i.e. adenine-thymine and cytosine-guanine.

**Figure 5:**
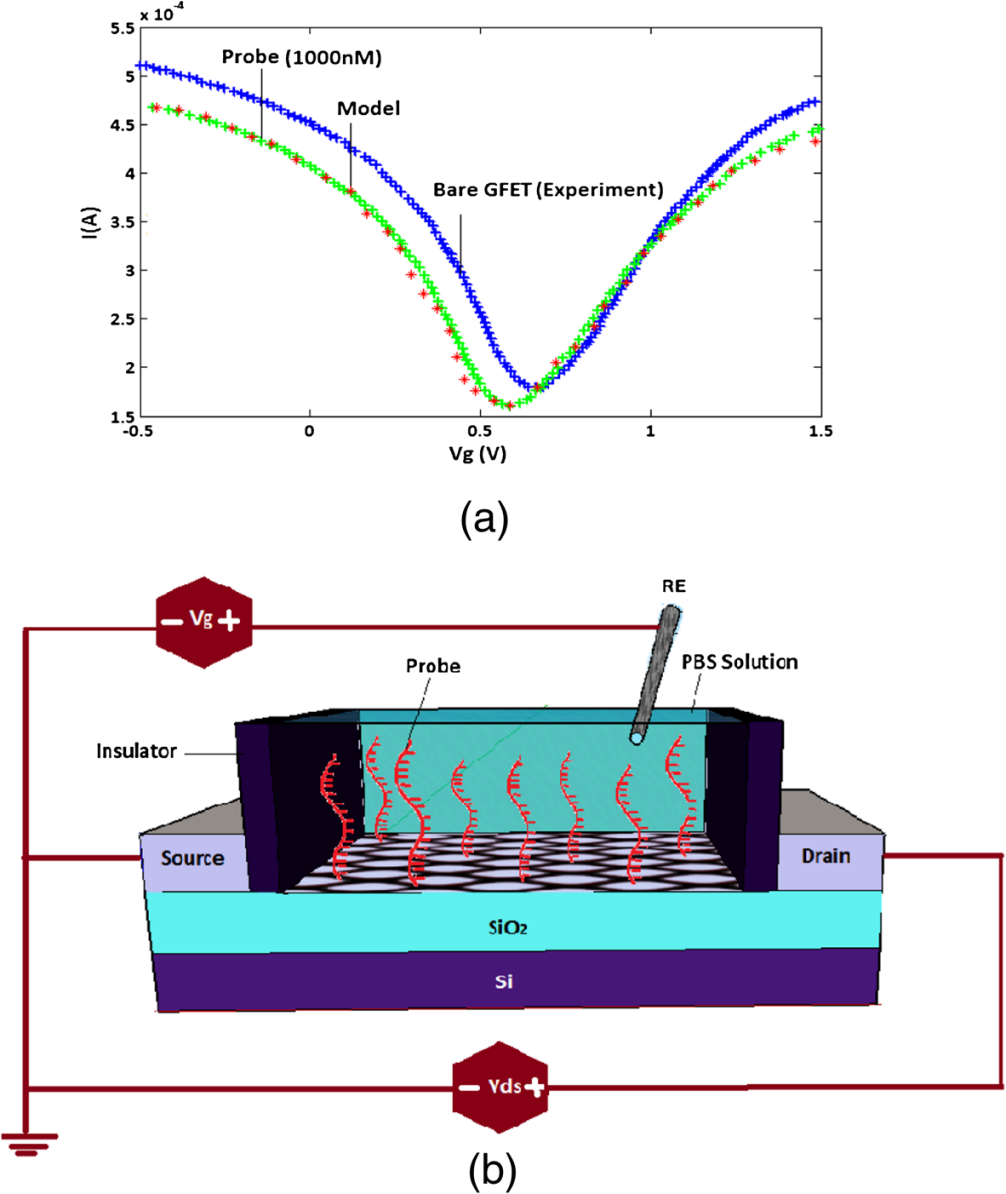
**The first step of hybridization detection concept. (a)** Comparison between SGFET-based DNA sensor model with extracted experimental data without adding DNA molecules (bare sensor) and after adding probe DNA. **(b)** Schematic of probe immobilization in SGFET.

There are still inadequate experimental results and accurate theoretical models of SGFET devices incubated in DNA solutions which are able to explain their detection mechanism and source of the experimentally observed signal generation. In this paper, SGFET-based optimized models are employed as detectors of DNA immobilization and hybridization. The proposed model describes the behaviour of the SGFETs device to detect the hybridization of target DNAs to the probe DNAs pre-immobilized on graphene with capability to distinguish single-base mismatch. The methodology of this study is presented for diagnosis of the SNP which uses an optimized model of graphene-based DNA sensor. This detection concept starts with showing the current-voltage characteristic of the SGFET-based DNA sensor before adding any DNA molecule (bare sensor), as shown in Figure 5. In the experiment, the SGFET devices must be washed with (40 µL) phosphate buffer (PB) to measure the dependence of conductance versus gate voltage [6]. Next step is continued by assuming that our optimized model is capable of differentiating between complementary and single-based mismatched DNAs which is an important characteristic with regard to the analysis of mutations and polymorphisms [49]. To address this possibility, SGFETs devices have been exposed to the ssDNA capture probes [50].

As shown in Figure 5, by applying the gate voltage to the DNA solution, it is obviously affirmed that the conductance of SGFET shows amipolar behaviour since the Fermi energy can be controlled by the gate voltage. Based on this outstanding characteristic, it is notable that the graphene can continuously be switched from the p-doped to the n-doped region by a controllable gate voltage. At the transition point where the density of electron and hole are the same, the minimum conductance (*V*_gmin_) is detected. This conjunction point is called charge neutrality point (CNP). The doping states of graphene have been monitored by the *V*_g,min_ to measure the minimum conductance of the graphene layer which is identified from the transfer characteristic curve.

It can be seen in Figure 5 that by immobilization of the probe DNAs, either complementary or mismatch, on the graphene surface, the *V*_g,min_ is considerably left-shifted by 10 mV. This fact can demonstrate the dependency of *V*_g,min_ on the immobilization of the probe DNA and hybridization of the complementary target DNAs. In other words, DNA molecules as n-dopants, shift the gate voltage leftwards due to the fact that DNA molecules n-dopes the graphene layer [6]. By introduction of DNAs as electron-rich molecules, the number of carriers would change in the graphene channel which has led in varying the conductance of source and drain [51-53]. SGFETs with high sensitivity is applied to detect the DNA hybridization based on the conductance variations. Finally, the hybridization event has been performed by introducing complementary sequences which include the target sequence of the probe DNA immobilized graphene device [54].

**Figure 6:**
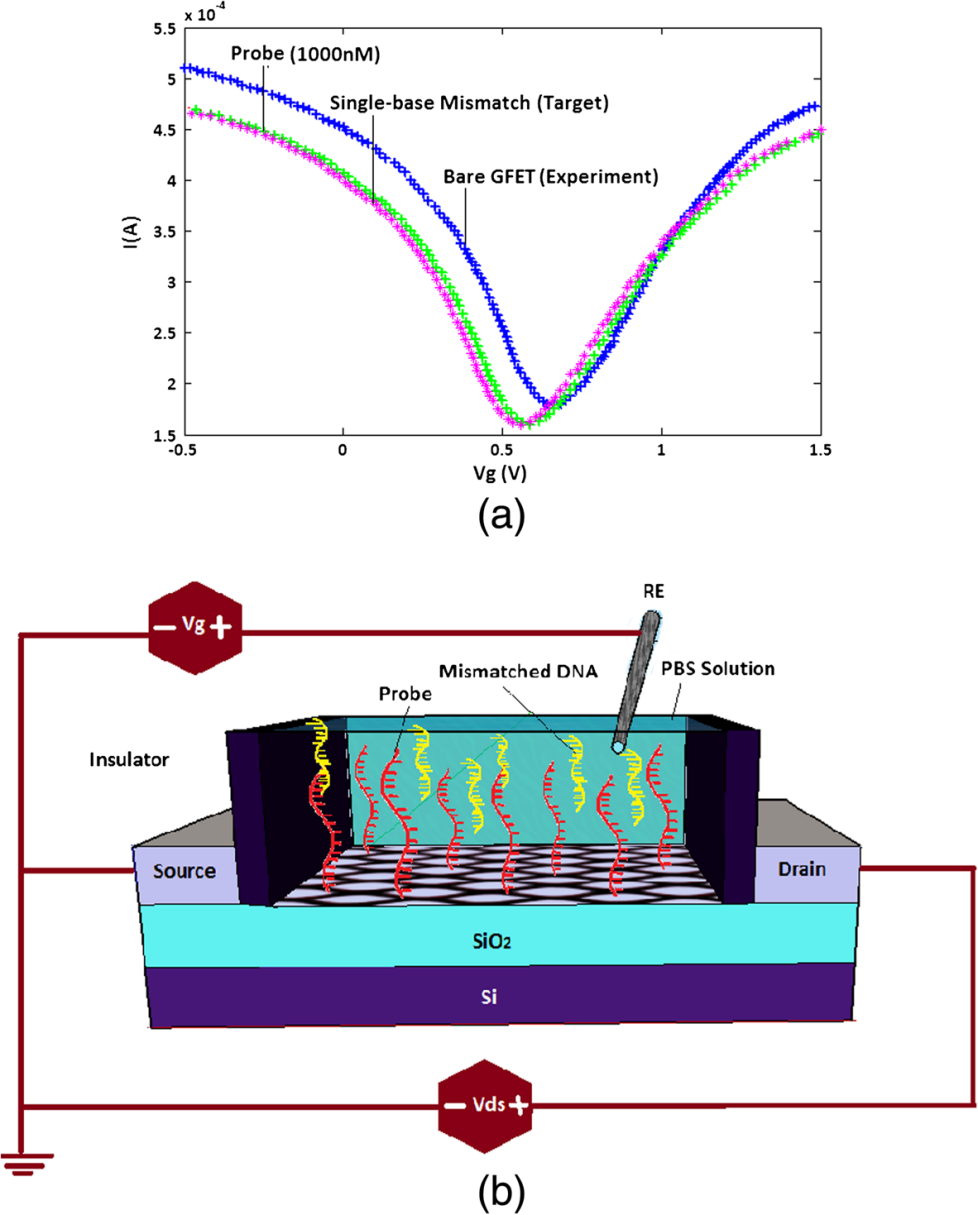
**Immersing the device in mismatched DNA solution. (a)** Conductance versus gate voltage curves after incubation with probe and; **(b)** after immersing the device in mismatched DNA solution.

As illustrated in Figure 6, the electronic responses of the SGFETs upon single-stranded DNA immobilization are compared with experimental results of subsequent DNA hybridization events [55]. Fascinatingly, single-base mismatch combination is occurring with the introduction of the non-complementary DNAs to the immobilized capture probe on SGFET device which results in no significant change in device characteristic which means conductance will be remained unchanged in this case. When the probe molecules expose to the target which is a mismatched DNA (non-complimentary) in this step, there is no bonding reaction between two pairs of DNA strands since they cannot hybrid because of the presence of mismatched base pair as illustrated in Figure 4. So there are no associated charges with the target molecule that can impose an obvious change to the applied gate voltage. It can also be seen that the SGFET device specifically recognizes the target DNA sequences. In light of this fact, the focus of this paper is to present a new strategy for DNA sensor with the capability of detection of SNP. According to the optimized model of SGFET-based DNA sensor using PSO algorithm, by substituting *α* = 2.138*e*^10^*F*^2^ + 8.9921*e*^9^*F* - 5.680*e*^3^ in Equation 1, the current-voltage characteristic of DNA sensor for detection of probe (*F* = 1, 000 nM) is:

(8) $$\begin{array}{ll} I_{d}= & \frac{3q^{2}{\left(3\pi a^{3}t^{3}k_{B}T\right)}^{0.5}}{\text{hL}}\left[{ȷ}_{-0.5}(\eta )+{ȷ}_{-0.5}(-\eta )\right]\\ & \times \;\;\left(\frac{2.138e^{10}F^{2}\;+\;8.9921e^{9}F\;-\;5.680e^{3}}{F}V_{\text{gs}(\text{without DNA})}\;-\;V_{t}\;\;\right) \end{array}$$

**Figure 7:**
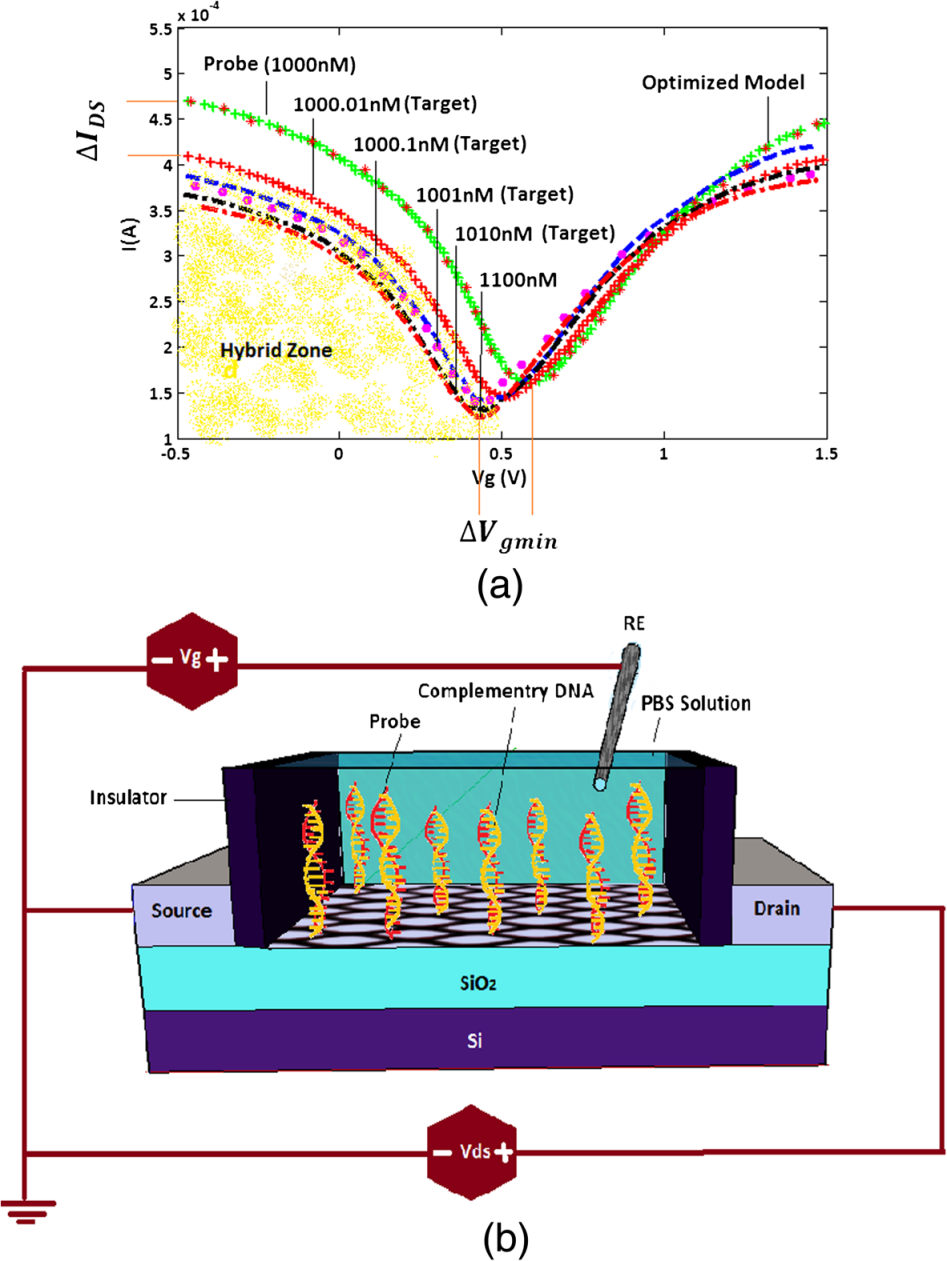
**The second step of hybridization detection concept. (a)** Conductance versus gate voltage of the SGFETs device after immersing in different concentrations of complementary DNA solution. **(b)** Schematic of hybridization event and forming fully matched DNA.

By employing the abovementioned equation, the *I*_*d*_-*V*_*g*_ characteristic of the optimized model is illustrated in Figure 5 and an acceptable agreement with the experimental data extracted from reference [49] is achieved. Figure 7 describes the *I*_*d*_ - *V*_*g*_ characteristic of the proposed model as well as the relevant experimental data for different concentrations of complementary DNA, where each diagram depicts specific concentration of the DNA molecules.

According to the experimental data, two important factors as detective parameters (*I*_ds_,*V*_gmin_) play crucial roles in detecting the DNA hybridization events. Whenever complementary DNA molecules are introduced to the sensor, these parameters will vary and decision will be made based on these variations. Table 3 can give us an idea about how *I*_ds_ and *V*_gmin_ parameters change with different concentration of complementary DNA molecules which reveals the sensitivity of *V*_g,min_ towards the hybridization of the target DNAs.

**Table 3:** ***I***_**ds**_**, *V***_**gmin **_**for different concentration of DNA molecules**

**Concentration *F *** **(nM)**	***V***_**gmin**_	***I***_**ds**_
*F*=1,000 (Probe)	0.54	4.7
*F*=1,000.01 (Target)	0.5	4.1
*F*=1,000.1 (Target)	0.45	3.98
*F*=1,001 (Target)	0.41	3.8
*F*=1,010 (Target)	0.40	3.7
*F*=1,100 (Target)	0.40	3.6

It is apparently seen that the considerable decrease of conductance is a sign of probe-target matching combination in DNA hybridization. The experimental data indicates the strong dependency of the gate voltage on the concentration increment which can have a predictable influence on the current-voltage characteristics of SGFET device. In other words, the *I*_*d*_ shifts downwards while the gate voltage shifts leftwards.

The complementary DNAs also successfully attach to the graphene surface through graphene-nucleotide interaction and impose n-doping effect which results as the left shift of *V*_g,min_ after DNA hybridization. It is stated that the stacking interaction between nucleotide and graphene surface upon DNA hybridization has a strong influence on *V*_g,min_, which can shift it leftwards [52]. This phenomena describes that the transfer of electrons from the target DNA happens because the probe DNA brings it to the proximity of the graphene surface [6]. In addition to the *V*_g,min_ shift, the *I*_*d*_ experiences a current decrease from 4.7 to 4.1 amp at *V*_g_ = -0.5*v*. Furthermore, when DNA molecule is present, the *I*_*d*_ continues to decrease with concentration increment of complementary DNAs. This fact can be explained by the p-type behaviour of graphene in the FET structure as observed by [56-59], which can justify the current decrease upon DNA hybridization event. While graphene is known as a p-type semiconductor with the holes as a majority of carriers, the electrons from DNA will lower the carrier concentration of graphene and hence reduce the conductance. By increasing the amount of complementary DNA concentration, more DNAs will make the configurational change and cause more electrons being trapped on the surface. The current or conductance shows a steady drop off at *V*_*g*_ = -0.5*v*.

Similar results had been reported for unfunctionalized graphene [59], where a larger current decrease was observed. The amount of shift rises with the increasing concentration of the complementary DNA from 1 to 10 nM as stated by experimental data [60]. The amount of these changes would determine that the hybridization event occurred in the presence of complementary or non-complementary DNA. For clarifying this condition, $\Delta I_{D\text{min}}^{\text{min}}$ and $\Delta V_{\text{gmin}}^{\text{min}}$ are introduced as the representatives of the source-drain current at voltage of *V*_gmin_ = -0.5*v* and the gate-voltage changes during hybridization events, respectively. The following equations describe the selected parameters:

(9) $$\Delta I_{D\text{min}}^{\text{min}}=|I_{D\text{probe}}-I_{\text{DF}=1000.01}|=0.6$$

(10) $$\Delta V_{\text{gmin}}^{\text{min}}=|V_{\text{gmin}\;\text{probe}}-V_{\text{gmin}\;F=1000.01}|=0.04$$

where *I*_*D*probe_ is the drain current of probe DNA molecule, *I*_DF_ denotes drain current in a specific DNA concentration, *V*_gmin probe_ represents the minimum gate voltage of probe DNA molecule while *V*_gmin *F*_ shows its concentration. The experimental data has to be obtained from the sample. In the next step, detective parameters should be extracted (*V*_gmin probe_, *I*_ds_|_Vgs = -0.5_) for probe and target DNA as well to calculate the *ΔI*_min_ and *ΔV*_gmin_ values. To make a decision from the obtained results, Table 4 is prepared and can be utilized.

**Table 4:** Decision making table based upon different conditions happened to detective parameters

**Conditions**	**Decision**
$\Delta V_{\text{gmin}}\geq \Delta V_{\text{gmin}}^{\text{min}}$ and $\Delta I_{\text{min}}\geq \Delta I_{\text{min}}^{\text{min}}$	Hybridization is happened
$\Delta V_{\text{gmin}}\geq \Delta V_{\text{gmin}}^{\text{min}}$ and $\Delta I_{\text{min}}\leq \Delta I_{\text{min}}^{\text{min}}$	Try again
$\Delta V_{\text{gmin}}\leq \Delta V_{\text{gmin}}^{\text{min}}$ and $\Delta I_{\text{min}}\geq \Delta I_{\text{min}}^{\text{min}}$	Try again
$\Delta V_{\text{gmin}}\leq \Delta V_{\text{gmin}}^{\text{min}}$ and $\Delta I_{\text{min}}\leq \Delta I_{\text{min}}^{\text{min}}$	SNP occurred

## Conclusion

Due to the outstanding properties of graphene nanomaterial such as high surface area, electrical conductivity and biocompatibility, it has remarkable potential for DNA and protein detection as a biosensing material. The detection of DNA hybridization is currently an area of intense interest whereas recent studies have proved that the mutations of genes are responsible for numerous inherited human disorders. In this research, graphene is chosen as both a sensing layer and a conducting channel in solution-gated field effect transistors for detection of DNA hybridization. In order to facilitate the rational design and the characterization of these devices, a DNA sensor model using particle swarm optimization theory developed and applied for detection of DNA hybridization. Furthermore, our proposed model is capable of detecting the single-nucleotide polymorphism by suggesting the detective parameters (*I*_ds_ and *V*_gmin_). Finally, the behaviour of solution-gated field effect transistor-based graphene is compared by the experiment results. An accuracy of more than 98% is reported in this paper which guarantees the reliability of an optimized model for any application of the graphene-based DNA sensor such as diagnosis of genetic and pathogenic deseases.

## Competing interests

The authors declare that they have no competing interests.

## Authors’ contributions

HK designed and performed the device modeling and simulation work, analyzed the data, and drafted the manuscript. RY and MTA supervised the research work. RR assisted with the optimization and proofread the manuscript. HH consulted in bio-molecular studies and assisted in analyzing DNA behaviors. All authors read and approved the final manuscript.
